# Morning blood pressure surge in the early stage of hypertensive patients impacts three-dimensional left ventricular speckle tracking echocardiography

**DOI:** 10.1186/s40885-021-00173-3

**Published:** 2021-08-15

**Authors:** Ami Kwon, Sang Hyun Ihm, Chan Seok Park

**Affiliations:** 1 Division of Cardiology, Department of Internal Medicine, Seoul St. Mary’s Hospital, College of Medicine, The Catholic University of Korea Seoul, Seoul, Republic of Korea; 2 Division of Cardiology, Department of Internal Medicine, Bucheon St. Mary’s Hospital, College of Medicine, The Catholic University of Korea Seoul, Seoul, Republic of Korea

**Keywords:** Blood pressure, Ambulatory blood pressure monitoring, Three dimensional echocardiography, Speckle tracking echocardiography, Left ventricular deformation

## Abstract

**Background:**

The aim of this study was to examine left ventricular (LV) function in untreated, newly diagnosed hypertensive patients with morning blood pressure surge (MBPS) status using three-dimensional (3D) speckle tracking echocardiography (STE).

**Methods:**

In this study, 163 newly diagnosed hypertensive patients were included, and all patients underwent 24-h ambulatory blood pressure monitoring (ABPM). According to ABPM, participants were divided into a MBPS group and a non-MBPS group. The entire study population was examined by complete two-dimensional (2D) transthoracic echocardiography (TTE) and 3D STE.

**Result:**

The results of this study showed that 3D LV longitudinal strain was significantly decreased in the MBPS group compared with the non-MBPS group (− 30.1 ± 2.0 vs. -31.1 ± 2.7, *p* = 0.045). Similar trends were observed for 3D twist (9.6 ± 6.1 vs. 12.1 ± 4.8, *p* = 0.011) as well as for 3D torsion (1.23 ± 0.78 vs. 1.49 ± 0.62, *p* = 0.042). The LV principal strain was decreased in the MBPS group (− 33.9 ± 1.7 vs. -35.5 ± 2.8, *p* < 0.001). The 3D LV global longitudinal strain (GLS) and principal strain were significantly associated with quartile of MBPS as measured by systolic blood pressure (SBP).

**Conclusion:**

The 3D STE revealed that LV mechanics were more impaired in the MBPS group than in the non-surge newly diagnosed, untreated hypertensive patients; even the 2D TTE parameters showed no difference.

## Background

Hypertension (HBP) is considered a major risk factor for cardiovascular diseases, especially fatal or non-fatal stroke and coronary events, with a peak incidence in the morning previously reported [1–3]. Some studies showed the association between with morning blood pressure surge (MBPS) and subsequent cardiovascular complications, both in hypertensive patients [1, 4–7] and the general population [8–10]. By dividing MBPS degree by quartile, MBPS was positively associated with LV mass index. Moreover, MBPS is associated independently with left ventricular hypertrophy (LVH) as target organ damage (TOD) of the heart [11]. An exaggerated MBPS is associated with echocardiographic measures of hypertensive heart disease. MBPS increases cardiac after-load and arterial stiffness, contributing to progression of LVH [12].

To investigate the effect of HBP on LV structure and function, electrocardiogram and conventional 2D echocardiography are widely performed in clinical practice. Echocardiography is an accurate and quantitative method to evaluate for changes of heart function and structure by HBP. LVH and diastolic dysfunction are hypertensive heart insults that can be assessed by TTE, but conventional 2D TTE has limitations to detect early hypertensive cardiac changes. Further, recent studies have investigated that patients with high normal blood pressure and arterial hypertension have subclinical myocardial dysfunction even before development of overt LVH [13, 14].

STE can provide mechanical insights into LV systolic function to detect early cardiac dysfunction before abnormalities can be observed with traditional LV function measurements [15]. Recent advances in 3D STE technique with novel strain parameters have led to more accurate and reproducible results than previous conventional 2D TTE. As LV deformation is 3D, three strain values (longitudinal, circumferential, and radial) are used, and LV rotational parameters, such as torsion and twist with shear deformation, help define deformations in LV. Moreover, principal strain is a method for describing multi-dimensional deformations that is widely applied in structural change applications [16]. Principal strain is applied well for biologic tissues with an underlying structure of muscle fibers, and strain direction can be related to actual fiber direction. Therefore, application of these novel strains to myocardial deformation can be useful in characterizing effective LV systolic function and underlying structural changes [17, 18].

The objective of the study was to assess the presence and extent of hypertensive changes of LV myocardial function, as well as mechanics in individuals with newly diagnosed, never-treated hypertension according to MBPS status on 3D STE.

## Methods

### Participants

Between November 2016 and October 2019, we recruited 182 newly diagnosed and untreated hypertensive patients aged from 18 to 80 years. Participants with diabetes mellitus needing oral medication or insulin therapy; secondary hypertension diagnosed during the initial workup; current or previous history of significant arrhythmia, such as atrial fibrillation, left ventricular ejection fraction < 50%, evidence of major valvular heart disease (i.e., any degree of mitral or aortic stenosis; greater than mild degree of aortic, mitral, or tricuspid regurgitation; and presence of a prosthetic valve); greater than mild pericardial effusion; and poor echocardiographic window were excluded from the study.

Laboratory tests were obtained from all the participants included in the study. Abdominal circumference (AC) was measured for each patient. AC is relatively easy to measure in the office without special equipment and were significantly positively associated with metabolic syndrome. The study was approved by the local ethics committee, and informed consent was obtained from all patients.

### Ambulatory BP monitoring

Ambulatory blood pressure monitoring (ABPM) was performed with a portable non-invasive recorder (TM-2439, A&D Medical, Tokyo, Japan) on a day of usual activity. At each reading, participants were instructed to remain motionless and to record their activity on a diary sheet. Technical aspects have been previously reported [19]. Ambulatory BP readings were obtained at 15-min intervals from 6 am to midnight and at 30-min intervals from midnight to 6 am. Recordings were automatically edited if SBP > 260 or < 70 mmHg or if DBP > 150 or < 40 mmHg and pulse pressure > 150 or < 20 mmHg. Participants had recordings of good technical quality (at least 70% valid readings). Nighttime BP was defined as the average BPs from the time when the subject went to bed until the time the subject got out of bed, and daytime BP was defined as the average BPs recorded during the rest of the day. Awakening BP was defined as the average BPs during the first 2 h after wake-up time, 4 times of BP readings. MBPS was defined as a sleep-trough surge, as morning BP (two-hour average of four 30-min BP readings just after waking) minus the lowest nocturnal BP (one-hour average of the three BP readings centered on the lowest nighttime reading). We subclassified the patients according to extent of the sleep-trough MBPS as follows: top decile of sleep-trough MBPS (> 35 mmHg; the morning surge group) versus all other individuals (the non-MBPS group). We divided the entire study population into quartiles of MBPS of SBP. With these quartiles, we evaluated the differences in 3D STE values.

### Echocardiography

Echocardiographic examinations were performed using commercially available Vivid E9 (GE Healthcare, Milwaukee, WI) and Epiq 7 (Philips Healthcare, Andover, MA) ultrasound machines equipped with a 2.5 MHz transducer and a 4 V-D and xMATRIX phased array transducer, respectively, for 3D data set acquisitions.

### 2D standard echocardiographic examination and Doppler tissue imaging (DTI)

Echocardiographic images were obtained according to the standard guideline recommended by the American Society of Echocardiography [20]. Left ventricular end-systolic and end-diastolic dimensions, posterior wall, and septal thickness were measured according to the current guidelines. LV end-systolic and end-diastolic volumes were measured using the modified biplane Simpson’s method from apical four- and two-chamber views, and LVEF was calculated by the same method. LA volume was measured in LV end-systole by the Simpson’s biplane method in apical two- and four-chamber views.

The mitral early diastolic flow (E) and late diastolic flow (A) velocities were acquired, and the E/A ratio was calculated. The deceleration time of the mitral E wave also was measured. Doppler tissue imaging was obtained from apical four-chamber view. The septal and lateral wall tissues were measured using pulse-wave Doppler tissue imaging at the septal and lateral mitral annulus and were averaged (over three-beat recording assessments) to obtain the mean early diastolic mitral annular velocity and the ratio of early diastolic transmitral flow velocity to early diastolic mitral annular velocity.

### 3D echocardiographic examination and speckle tracking analysis

The 3D echocardiographic images were obtained in single-beat mode at a rate of 24 ± 6 frames/sec; to achieve optimal spatial resolution, the pyramidal scan volume was focused on the LV chamber to include the entire LV. Six electrocardiogram-gated consecutive beats were acquired during a single breath hold.

All datasets were transmitted to and analyzed by a commercially available system (4D LV-analysis Version 3.0; TomTec Imaging Systems, Unterschleissheim, Germany). The software automatically identified the LV endocardial surface of the three apical views (four-, three-, and two-chamber views). The 3D strain values were calculated as the average of the regional values from the 17 myocardial segments at end-systole. Technical details of the calculation process for principal strain and rotation were previously published [16, 18].

### Reproducibility analysis

To evaluate intra-observer variability in the offline analysis, 10 subjects were randomly selected and analyzed by the same operator with at least a 1-week interval between the two analyses. To assess the effect of inter-observer variability, the same 10 patients were analyzed in a random order at different times using the same software by a second investigator who was blinded to the results from the first investigator.

### Statistical analysis

Statistical analysis was performed using commercially available software (MedCalc version 14.10.02; MedCalc Software, Ostend, Belgium). All continuous variables were shown as the mean ± standard deviation (SD). Differences in continuous variables between the surge and non-surge states were estimated using the paired t-test and one-way analysis of variance (ANOVA). The Chi-square test was applied to assess differences between categorical variables. Visual descriptions of the changes in 3D strain parameters with surge status and by quartile were illustrated using the box-and-whisker plot method. *P*-values < 0.05 were considered statistically significant.

## Results

A total of 182 patients was enrolled in this study, after 19 individuals were excluded for the following reasons: seven had insufficient ABPM, six had arrhythmia during an echocardiographic examination, five had LVEF less than 50%, and one had a poor echocardiographic window. All enrolled participants underwent 24-h ABPM and were divided into two groups according to 24-h SB*P* value: 33 individuals with MBPS and 130 with non-MBPS.

Baseline characteristics and laboratory findings of the study population by MBPS status are summarized in Table 1. The mean age was significantly higher in MBPS patients. Individuals with or without MS did not differ in sex, abdominal circumference, and laboratory findings except triglyceride level.

**Table 1:** Baseline characteristics of the study participants

	Surge (*n* = 33)	Non surge (*n* = 130)	*P* value
Age (years)	53 ± 13	47 ± 14	*0.043*
Male (%)	14 (42)	80 (62)	0.074
Abdominal circumference (cm)	92.9 ± 8.6	92.5 ± 9.5	0.860
Hemoglobin (g/dL)	14.4 ± 1.5	14.7 ± 1.6	0.271
Fasting Blood Glucose (mg/dL)	99.8 ± 11.5	104.7 ± 25.4	0.105
BUN (mg/dL)	13.1 ± 3.2	13.7 ± 3.8	0.401
Creatinine (mg/dL)	1.06 ± 1.223	0.81 ± 0.191	0.254
Protein (g/dL)	7.4 ± 0.3	7.3 ± 0.5	0.323
Albumin (g/dL)	4.6 ± 0.3	4.7 ± 0.4	0.068
Sodium (mEq/L)	141.6 ± 1.9	141.6 ± 9.5	0.843
Potassium (mEq/L)	4.2 ± 0.5	4.3 ± 0.4	0.572
AST (U/L)	25.2 ± 13.8	24.6 ± 12.2	0.791
ALT (U/L)	31.8 ± 24.7	32.5 ± 26.5	0.882
Total Cholesterol (mg/dL)	200.7 ± 39.6	201.6 ± 48.1	0.920
Triglyceride (mg/dL)	124.9 ± 63.0	175.8 ± 101.9	*< 0.001*
HDL (mg/dL)	55.7 ± 13.9	51.0 ± 14.9	0.108
LDL (mg/dL)	136.0 ± 39.4	134.1 ± 41.0	0.814
Uric acid (mg/dL)	5.7 ± 1.3	5.6 ± 1.6	0.936

Data are mean ± SD or percentage as marked

*BUN* blood urea nitrogen, *AST* aspartate aminotransferase, *ALT* alanine aminotransferase, *HDL* high-density lipoprotein, *LDL* low-density lipoprotein

As defined, the mean awaken BP was significantly higher in the MBPS group than in the non-MBPS group (Table 2). Although there were no significant differences in daytime and nighttime BPs between the two groups, nighttime standard deviations (SDs) of SBP and diastolic BP were significantly higher in the MBPS group. The mean ± SD sleep-trough MBPS defined by the difference between morning SBP and lowest nocturnal SBP was 29.5 ± 10.6 mmHg for the MBPS group and 8.3 ± 7.4 mmHg for the non-MBPS group.

**Table 2:** ABPM characteristics

	Surge (*n* = 33)	Non surge (*n* = 130)	*P* value
Daytime			
SBP	156.8 ± 11.4	156.4 ± 14.4	0.875
SBP, SD	15.7 ± 4.5	14.9 ± 4.2	0.248
DBP	100.3 ± 12.5	102.6 ± 12.4	0.338
DBP, SD	13.0 ± 3.5	12.2 ± 4.2	0.327
HR	75.6 ± 7.8	78.6 ± 11.0	0.148
HR, SD	10.4 ± 5.1	11.1 ± 5.2	0.476
Nighttime			
SBP	148.1 ± 16.3	146.8 ± 16.1	0.691
SBP, SD	15.8 ± 4.6	12.6 ± 4.1	*< 0.001*
DBP	89.8 ± 19.9	94.1 ± 13.1	0.139
DBP, SD	12.6 ± 5.0	10.4 ± 3.0	*0.004*
HR	66.3 ± 9.6	68.1 ± 10.2	0.380
HR, SD	6.8 ± 3.7	7.7 ± 5.1	0.353
Awakening			
SBP	160.1 ± 18.0	144.3 ± 17.3	*< 0.001*
SBP, SD	12.6 ± 5.9	9.0 ± 4.7	*< 0.001*
DBP	101.4 ± 16.4	92.7 ± 14.1	*0.003*
DBP, SD	10.9 ± 7.8	7.7 ± 4.1	*0.001*
HR	65.7 ± 10.6	65.2 ± 9.6	0.774
HR, SD	7.5 ± 6.3	4.4 ± 3.0	*< 0.001*
BP differences	29.5 ± 10.6	8.3 ± 7.4	*< 0.001*

Data are mean ± SD or percentage as marked

*SBP* systolic blood pressure, *SD* standard deviation, *DBP* diastolic blood pressure, *HR* heart rate

### 2D and 3D echocardiographic analyses

Table 3 shows the parameters for LV function in 2D and 3D TTE depending on the presence or absence of MBPS. The 2D LV dimensions, volumes, wall thickness, and diastolic function were similar between the two groups, whereas the mean value of LV end-diastolic volume was significantly larger in the MBPS group. LVEF in 2D and 3D methods had similar values in the two groups.

**Table 3:** Echocardiographic parameters of the study population

	Surge (*n* = 33)	Non Surge (*n* = 130)	*P* value
2DE parameters			
Septal thickness (mm)	10.0 ± 1.4	10.2 ± 1.7	0.691
Posterior wall thickness (mm)	9.4 ± 1.5	9.6 ± 1.6	0.497
LV end-diastolic dimension (mm)	47.2 ± 4.2	47.1 ± 4.3	0.880
LV end-systolic dimension (mm)	29.1 ± 4.7	29.0 ± 4.5	0.931
LV end-diastolic volume (mL/m^2^)	53.8 ± 10.1	50.0 ± 8.8	*0.033*
LV end-systolic volume (mL/m^2^)	19.2 ± 5.4	17.8 ± 4.2	0.155
LVEF (%)	63.9 ± 3.9	64.2 ± 4.2	0.746
LV mass index (g/m^2^)	94.3 ± 15.8	94.9 ± 21.3	0.878
E wave (cm/s)	64.3 ± 15.4	67.5 ± 16.8	0.319
A wave (cm/s)	72.0 ± 20.3	72.9 ± 15.6	0.801
EA ratio	0.94 ± 0.33	0.98 ± 0.35	0.714
Deceleration time (ms)	215.9 ± 46.6	206.1 ± 42.3	0.245
LA volume index (mL/m^2^)	36.3 ± 5.1	36.4 ± 5.4	0.904
Septal e′ velocity (cm/s)	7.18 ± 2.07	7.61 ± 2.17	0.305
Septal E/e′	9.42 ± 2.78	9.48 ± 2.75	0.913
Lateral e′ velocity (cm/s)	10.08 ± 2.88	9.75 ± 2.57	0.523
Lateral E/e′	6.90 ± 2.60	7.25 ± 2.13	0.428
Average of septal and lateral E/e′	7.87 ± 2.57	8.10 ± 2.28	0.617
3DE			
End-diastolic volume (mL)	94.1 ± 20.2	91.9 ± 19.1	0.565
End-diastolic volume index (mL/m^2^)	52.2 ± 8.3	51.2 ± 8.8	0.568
End-systolic volume (mL)	35.8 ± 8.4	34.9 ± 8.6	0.590
End-systolic volume index (mL/m^2^)	19.9 ± 3.7	19.4 ± 4.1	0.578
Stroke volume (mL)	58.3 ± 12.4	57.0 ± 11.5	0.577
Ejection Fraction (%)	62.0 ± 2.4	62.2 ± 3.3	0.807

Data are mean ± SD or percentage as marked

*2DE* two dimensional echocardiography, *LV* left ventricular, *EF* ejection fraction, *LA* left atrium, *3DE* three dimensional echocardiography

### 3D LV STE

**Fig. 1:**
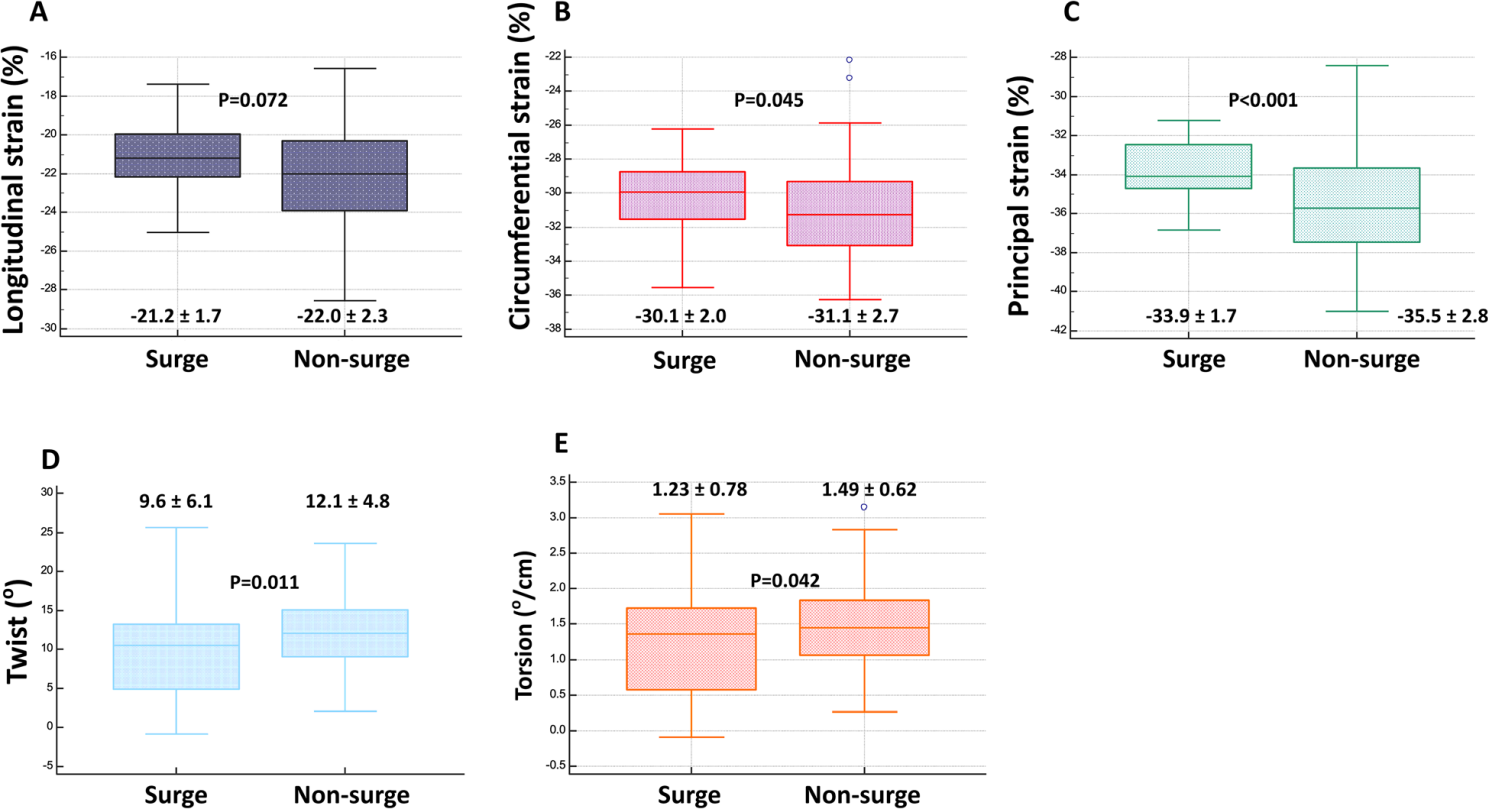
Box plot comparing three dimensional left ventricular strains in morning blood pressure surge and non-surge group: **A** Longitudinal strain, **B** Circulmferential strain, **C** Principal strain, **D** Twist, **E** Torsion

When comparing strains with or without morning surge, longitudinal strain showed a lower tendency in patients without morning surge, but there was no statistical difference. Other parameters, including circumferential strain, twist, torsion, and principal strain, were significantly decreased in the non-MBPS group (Fig. 1).

**Fig. 2:**
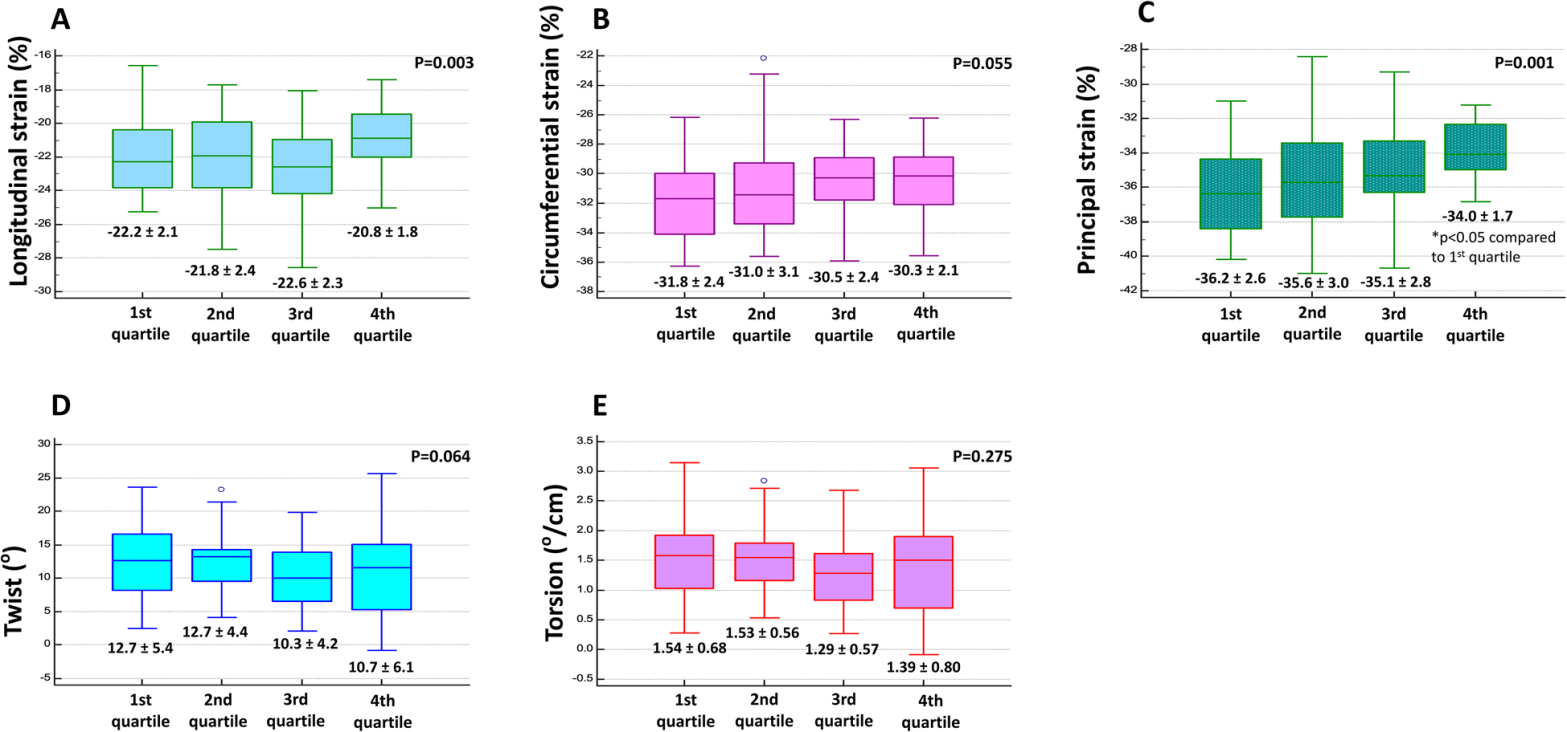
Morning blood pressure surge and three dimensional left ventricular strains according to the level of morning BP surge, the lowest quartile to highest quartile: **A** Longitudinal strain, **B** Circulmferential strain, **C** Principal strain, **D** Twist, **E** Torsion

When compared by quartile according to degree of morning surge, there was a significant difference only in longitudinal strain and principal strain groups. Other strain parameters (circumferential strain, twist, and torsion) showed no statistical difference (Fig. 2).

**Fig. 3:**
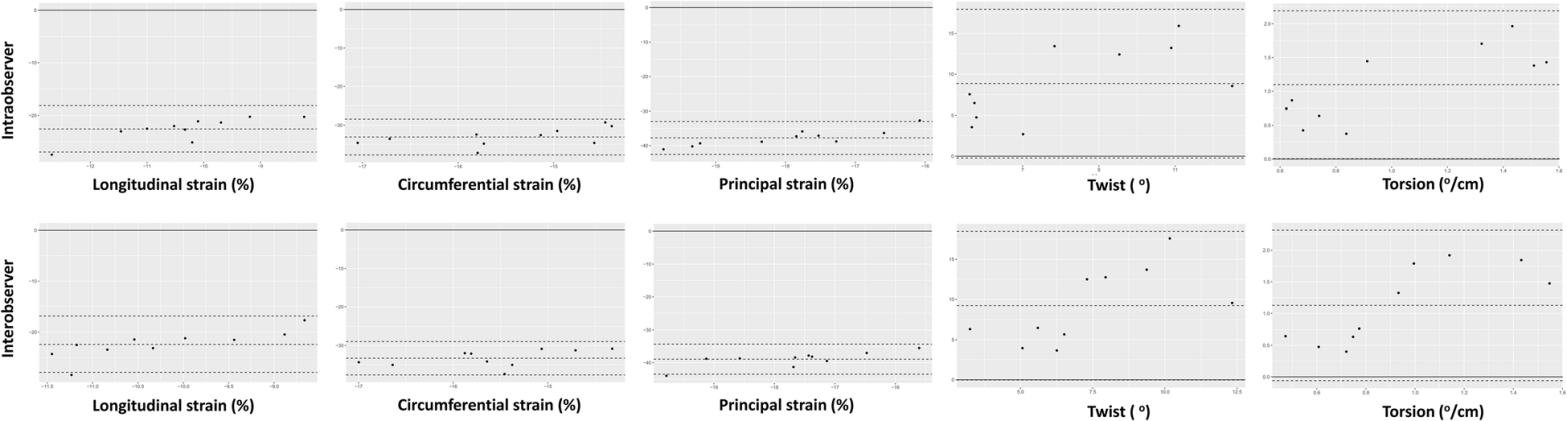
Bland-Altman analyses for intra- and inter-observer variability

All the measurements showed good agreement, and the Bland-Altman plots are presented in Fig. 3.

## Discussion

Our study showed that LV mechanics assessed by 3D STE, represented by longitudinal strain, circumferential strain, principal strain, twist, and torsion, is impaired in individuals with MBPS, while 2D conventional echocardiographic parameters show few differences. In addition, 3D LV longitudinal and principal strain, which represent LV deformation, tended to gradually worsen from the first quartile MBPS (lower MBPS difference in our study) to the fourth quartile, except third quartile of longitudinal strain.

Hypertension is associated with cardiac structural and functional changes, as well as increased frequency of cardiovascular events. MBPS is one of the components of diurnal BP variability, and normal MBPS is a physiological phenomenon. However, “exaggerated” MBPS is pathological and is expected to progress to TOD, to trigger serious cardiovascular events [21], and is an independent risk factor of stroke [1]. A high MBPS and associated factors could contribute to increased risk of coronary events. The role of peak SBP in predicting coronary events in older patients has been described [22]. Moreover, a higher MBPS is associated with this cardiovascular risk independent of the level of ABPM and nocturnal BP decrease. Since our study targeted patients with early-stage hypertension, we were able to examine pure cardiac changes by MBPS without interference from other factors related to HBP.

Clinically, 2D echocardiography is used to detect the TODs due to HBP, including LVH and diastolic dysfunction and in advanced stages both systolic and more worsened diastolic dysfunction. However, little is known about the LV mechanics in hypertensive patients with subclinical cardiac damage. Recent studies have shown that patients with high normal blood pressure and early systemic arterial hypertension have subclinical myocardial dysfunction before development of LV hypertrophy [13, 14]. Sometimes, diastolic function measured with DTI is only change in hypertensive cardiac damage and diastolic dysfunction [23, 24]. Doppler-based techniques can only measure velocities along the ultrasound beam, while velocity components perpendicular to the beam remain undetected [25]. Therefore, DTI has inherent limitations in measuring LV mechanics, which involves complex movements. In our study, no suggestion of hypertensive change in heart and no difference with or without MBPS in 2D DTI was noted.

STE provides an ability to detect early cardiac dysfunction in a variety of disease states before abnormalities can be observed with traditional LV function measurements [15]. Both DTI and STE measure motion against a fixed external point in space. However, STE has the advantage of being able to measure this motion in any direction within the image plane, whereas DTI is limited to the velocity component toward or away from the probe [25]. Strain and strain rate increase sensitivity in detecting subclinical cardiac involvement in hypertensive heart disease [26]. LV mechanics assessed by 2D speckle tracking imaging in arterial hypertension has been evaluated [27].

Longitudinal, circumferential, and radial LV systolic function assessed by 2D TTE were not equally damaged by increased SBP. In general, longitudinal LV mechanics, predominantly governed by the subendocardial layer, is most vulnerable and sensitive to the presence of myocardial disease. However, in transmural insults results with mid-myocardial and subepicardial dysfunction, reduction in LV circumferential and twist mechanics and in EF can be observed [26].

The LV rotation, represented as twist and torsion, is shown to be a marker of LV systolic [28, 29] and diastolic function [30]. This indicates that LV myocardial function in hypertensive patients is deteriorated during the entire cardiac cycle. Multiple recent studies have demonstrated that STE represents a clinically feasible alternative to TDI in evaluation of myocardial rotation and twist mechanics in the majority of patients [25, 31].

3D STE has been suggested to have a role by offering more precise measurements and circumventing the errors observed in 2D speckle-tracking-derived myocardial strains, such as use of foreshortened views that affect the accuracy of the individual component of myocardial motion. Further, the speckles of the 2D imaging plane might not always be valid because of the complex 3D motion of the myocardium that cannot be tracked in and out of the 2D image plane [25]. Moreover, 3D principal strain has proven to be effective in detecting subclinical cardiac damage [16, 32]. A previous study showed that, in patients with hypertension, principle strain analysis can be related to left ventricular myofiber geometry and can simplify assessment of 3D left ventricular deformation by circumventing the need to assess multiple shortening and shear strain components. Finally, the longitudinal and circumferential values were unchanged, though the pattern of contraction was altered [16].

Some studies have measured LV myocardial damage using DTI and 2D or 3D STE and have recognized TOD according to BP variability. According to Tadic et al., SBP changes in nighttime affects cardiac function, and there were differences between dippers and non-dippers in 2D GLS, twist, and all 3D strain parameters (GLS, global circumferential strain, radial strain, and global area strain) [33].

Our results are consistent with these findings that subclinical changes in LV mechanics can be detected using 3DE strains in hypertensive patients who had no changes in 2DE, although there was a statistical difference between the two groups in mean age known as the cause of diastolic dysfunction. LV systolic strain was higher in the non-surge group in comparison with the MS group.

The new finding of our study is that the longitudinal strain and principal strain of 3DE myocardial strains increased gradually and significantly from lower to higher MS quartile, whereas other parameters (circumferential strain, twist, and torsion) did not.

### Study limitations

There are several limitations in this study. This study was performed in a single center with a relatively small number of patients and no clinical follow-up. Further, there was no validation of our strain values with 2D STE or cardiac MRI. Each strain has its own reference value with participant age, but we did not conduct the study with age-matched individuals. In acquiring 3D images, there can be differences in the process depending on vendor, but such a difference might be negligible in our study since vendor-independent software was used. We did not exclude patients whose sleep was severely disturbed by earing the ABPM, all of our subjects showed nocturnal HBP with non-dipper pattern, we should have managed the quality of sleep more carefully. And only third quartile of longitudinal strain had different trend compared to other strain values, further studies were needed.

## Conclusion

This study revealed that 3DE-estimtated LV subclinical damage was increased in MS patients compared to the non-surge group. Longitudinal and principal strain were correlated with MS degree and worsened as MS degree increased, respectively. Our study could help to detect subclinical TOD by HBP earlier and more precise than previous modalities, such as ECG or 2D conventional TTE. Further longitudinal analyses with a large number of individuals are needed to evaluate subclinical cardiac damage in MS and its outcomes, as well as the benefit of therapeutic intervention in hypertensive patients.

## Data Availability

All authors have full control of all primary data, and they agree to allow the journal to review their data if requested. The datasets used and/or analyzed during the current study are available from the corresponding author upon reasonable request.

## Abbreviations

AC: Abdominal circumference
ABPM: Ambulatory blood pressure monitoring
BP: Blood pressure
DTI: Doppler tissue imaging
GLS: Global longitudinal strain
HBP: Hypertension
LV: Left ventricular
LVEF: Left ventricular ejection fraction
LVH: Left ventricular hypertrophy
MBPS: Morning blood pressure surge
SBP: Systolic blood pressure
SD: Standard deviation
STE: Speckle tracking echocardiography
TOD: Target organ damage
TTE: Transthoracic echocardiography
2D: Two-dimensional
3D: Three-dimensional
